# Neglect and perceived stigmatization impact psychological distress of orphans in Tanzania

**DOI:** 10.3402/ejpt.v6.28617

**Published:** 2015-11-19

**Authors:** Katharin Hermenau, Ina Eggert, Markus A. Landolt, Tobias Hecker

**Affiliations:** 1 Department of Psychology, University of Konstanz, Konstanz, Germany; 2 vivo international, Konstanz, Germany; 3 Department of Psychosomatics and Psychiatry, University Children's Hospital Zurich, Zurich, Switzerland; 4 Department of Psychology, University of Zurich, Zurich, Switzerland

**Keywords:** Orphans, maltreatment, neglect, mental health, stigmatization, sub-Saharan Africa

## Abstract

**Background:**

Research has shown that orphans in sub-Saharan Africa are at increased risk for mental health problems. Exposure to maltreatment and HIV/AIDS-related stigmatization are related to orphans’ psychological distress. Yet, researchers stress the need for more research in low-income countries to identify which factors of being an orphan may lead to psychological distress.

**Objectives:**

The present study aims to systematically investigate orphans’ experiences of maltreatment and stigmatization to identify factors that relate to their psychological distress.

**Methods:**

In total, 89 Tanzanian children who had lost at least one parent were compared to 89 matched non-orphans (mean age: 11 years; 51% boys). We measured exposure to maltreatment and perceived stigmatization as an orphan. Mental health was assessed using the Strengths and Difficulties Questionnaire, the Children's Depression Inventory, the UCLA PTSD Index for Children, and the Reactive–Proactive Questionnaire.

**Results:**

Orphans reported significantly more experiences of neglect, but not of abuse. A group comparison revealed more depressive symptoms, posttraumatic stress symptoms, and aggressive behavior among orphans. Neglect, abuse, and stigmatization correlated with orphans’ internalizing and externalizing problems, yet only neglect and stigmatization were related to orphans’ depression severity. Perceived stigmatization moderated the relationship between neglect and depression.

**Conclusions:**

Our findings suggest that orphans in Tanzania are at increased risk of experiencing neglect. Maltreatment and perceived stigmatization may play a role in orphans’ psychological distress. Culturally appropriate and evidence-based interventions may help to prevent maltreatment and stigmatization of orphans.

An orphan is defined as a child under the age of 18, who lost one or both parents due to death from any cause (UNICEF, 2006). The number of orphans is currently rising worldwide. Sub-Saharan Africa is the most affected region largely due to the HIV/AIDS-epidemic, with 56 million orphans in 2012 (UNICEF, 2014). For example, as of 2009 in Tanzania, one in four girls and one in five boys were orphans (UNICEF, 2011).

## Psychological consequences of parental loss

Secure attachment in early childhood plays a crucial role for psychological well-being across the lifespan (Fearon, Bakermans-Kranenburg, Ijzendoorn, Lapsley, & Roisman, 2010). Therefore, the death of a parental figure may strongly disrupt the attachment bonds and negatively impact the child's development. Concordantly, in nationally representative, Western community samples orphaned children were more likely than non-orphans to meet a diagnosis of any mental disorder (Fauth, Thompson, & Penny, 2009). In African countries, evidence has been found for orphans reporting higher depressive symptoms (Puffer et al., 2012; Unterhitzenberger & Rosner, 2014), more internalizing and externalizing problems (Atwine, Cantor-Graae, & Bajunirwe, 2005; Wild, Flisher, & Robertson, 2011), and greater posttraumatic stress disorder (PTSD) symptom severity than non-orphaned children (Cluver, Gardner, & Operario, 2007; Puffer et al., 2012).

Yet, research has shown that not all children develop psychological problems after the death of a parent (Stikkelbroek, Prinzie, de Graaf, Ten Have, & Cuijpers, 2012). Among the factors influencing whether a child develops disturbances are variables within the child and in the environment, such as quality of care for institutionalized children and the presence of additional stressors (Hermenau et al., 2011). Nyamukapa et al. (2010) showed that the association between being an orphan and psychological distress weakened after controlling for perceived stigmatization, inadequate care, child labor, and child abuse in Zimbabwean orphans.

## Exposure to maltreatment and its impact on orphans’ mental health

Though child maltreatment is a widespread phenomenon, studies focusing on abuse and neglect in low-income countries are very rare (Stoltenborgh, Bakermans-Kranenburg, Van Ijzendoorn, & Alink, 2013; Stoltenborgh, Bakermans-Kranenburg, & Van Ijzendoorn, 2013). It is even less common for such research to evaluate these experiences of orphans in low-income countries, even though approximately 70% of orphans in Tanzania reported physical abuse and about 30% reported emotional abuse during childhood (UNICEF, 2011). Yet, it is not clear whether orphans are systematically at higher risk of experiencing childhood abuse than their non-orphan peers. Qualitative studies have documented cases of the emotional and physical abuse of orphans (Morantz et al., 2013) and a national survey in Tanzania documented higher rates of emotional abuse for orphans, but not of physical abuse (UNICEF, 2011). Child abuse was equally common for orphans and non-orphans in Eastern Zimbabwe (Nyamukapa et al., 2010). A recent meta-analysis on orphans’ maltreatment in sub-Saharan Africa ascertained that orphans were not systematically at higher risk of experiencing physical abuse than non-orphans (Nichols et al., 2014). When focusing on neglect, however, orphans were more likely than non-orphans to go to bed hungry (Makame, Ani, & Grantham-McGregor, 2002), to be underweight (Miller, Gruskin, Subramanian, & Heymann, 2007), and to lack social support and basic needs (Puffer et al., 2012). Research on institutional childcare has documented poor child–caregiver ratios and inadequate caregiving as factors increasing the risk of physical and emotional neglect (Hermenau et al., 2011; Johnson, Browne, & Hamilton-Giachritsis, 2006).

Child maltreatment has been shown to be associated with a wide range of emotional and behavioral problems as well as psychiatric disorders (McLaughlin et al., 2012; Norman et al., 2012). Child maltreatment was also associated with earlier disorder onset, higher comorbidity and increased risk for depression in young adulthood compared to controls in a longitudinal design (Widom, DuMont, & Czaja, 2007), indicating the long-term effects of childhood adversities.

## Perceived stigmatization and orphans’ mental health

Stigma can be defined as the strong disapproval by society of an attribute held or perceived to be held by an individual or group (Chi, Li, Zhao, & Zhao, 2014). A meta-analysis identified a moderate mean correlation between various forms of stigmatization and average mental health scores in a sample of mostly western countries (Mak, Poon, Pun, & Cheung, 2007). In qualitative research, orphans reported experiences of stigmatization by different members of their society and attributed this stigmatization to their orphan status (Funkquist, Eriksson, & Muula, 2007; Morantz et al., 2013). Very few studies have assessed perceived stigmatization as an orphan. In one study, Atwine et al. (2005) ascertained that orphans reported more often that they felt treated differently than other children. Improved understanding of this subjective experience is critical, as research on HIV/AIDS-affected children reported contributory effects of HIV/AIDS-related stigmatization on mental health problems, particularly depression and other internalizing problems (Nyamukapa et al., 2010). When AIDS-related stigmatization and food insecurity were both present, likelihood of internalizing disorders increased substantially, indicating that stigmatization may moderate the negative relationship between neglect and orphans’ mental health (Cluver & Orkin, 2009). Further research is needed to understand the impact of stigmatization on mental health, particularly depression, and its interaction with other adverse experiences, specifically neglect.

## Objectives

Researchers have stressed the need for more studies in low-income countries to identify which factors of being an orphan may lead to psychological distress (Cluver & Gardner, 2007). Following prior findings (Puffer et al., 2012), we hypothesized 1) that orphans reported more neglect types but not more abuse types over their lifetime compared to matched non-orphans. Furthermore, we predicted 2) a higher level of psychological distress among orphans. Hypothesis 1) and 2) will be tested using multivariate analyses of variance (MANOVA).

We aimed to gain further insights about factors relating to orphans’ psychological distress. Therefore, in a second step we analyzed the group of orphans separately using regression analyses. We predicted 3) an incremental effect of perceived stigmatization due to their orphan status on externalizing and internalizing problems (including depressive symptoms) beyond the impact of maltreatment. As orphans are particularly at risk to experience neglect, we further hypothesized that 4) perceived stigmatization would moderate the relationship between neglect and internalizing and externalizing problems.

## Method

### Participants

We conducted a study at a primary school in a small town in southern Tanzania. All children from Class 2 to 7 were asked to take part. In total, we interviewed 409 children (52% boys) with a mean age of 10.49 years (SD=1.89, range: 6–15). The participation rate was 80%. For the present study, all children who had lost one or both parents were identified and matched to non-orphaned children according to sex and age. In case of more than one possible fit, the matching partner was selected randomly. Each group consisted of 89 children. The mean age of orphans was 11.09 years (SD=1.93, range: 6–15), and the mean age of non-orphans was 10.89 years (SD=1.72, range: 6–14). Groups did not differ in age [*t*(2, 176)=0.74, *p*=0.462]. In each group, 45 (51%) children were male. Of all orphans, 16 (18%) had lost both parents. Orphans who had lost both parents did not differ significantly from orphans who had lost one parent regarding reported maltreatment or mental health problems (Supplementary Table 1). The mean time since loss of the first parent was 6.56 years (SD=3.69). Fifty-three (60%) orphans had lived in foster care or in a children's home at one point in their life. Children who had lived in foster care or in a children's home did not differ from other orphans on any outcome parameter (Supplementary Table 2). In the full sample, the majority (77%; *n*=245) of the non-orphaned children had been living with both biological parents throughout their entire life. The parents of a minority of non-orphaned children (9%; *n*=28) were separated or divorced. Furthermore, some few non-orphaned children had been living with relatives, stepparents or in boarding schools, or other institutional care facilities (in Tanzania these facilities do not differ much from institutional care facilities for orphans regarding care quality). In order to compare orphaned children with average non-orphaned children and to control for rare influences, we included only non-orphaned children who had been living with both biological parents throughout their entire life in the analysis.

### Procedure

All structured interviews took place in a primary school in a small town in southern Tanzania. The research team consisted of five German psychologists and seven Tanzanian psychologists, and community workers. All staff members received a 2-week training session in skills for interviews with children. All measures were presented in interview format. A standardized form of assessment was practiced by all interviewers in joint and double-rated interviews. A high interrater-reliability was thus achieved (see below). Average interview time was 1.5 hours. All interviews were conducted individually in a private setting within the school grounds. Girls were interviewed by a female interviewer; children were assured that the interview was confidential and that they were free to end it at any time. By following established international guidelines (Brislin, Lonner, & Thorndike, 1973), all instruments were translated into Swahili and back to English in a blind, written form to assure accuracy of translation. Project leaders were present for the duration of the project and provided supervision for all staff members. Letters in English and Swahili were given to all caregivers to inform them about the objectives and procedure of the study. It was explained that participation would be entirely voluntary, that no monetary recompense would be given and that data would be analyzed anonymously. The Ethical Review Board of the University Konstanz, Germany and the Tanzanian Commission for Science and Technology approved the study. Other parts and aspects of the data gathered during the extensive investigation are presented in other recent reports (Hecker, Hermenau, Isele, & Elbert, 2014; Hermenau, Hecker, Elbert, & Ruf-Leuschner, 2014).

### Measures

At first, socio-demographic information, including age, class, and sex, was collected.

#### Maltreatment

Maltreatment was measured using the Maltreatment and Abuse Chronology of Exposure—Pediatric Version (pediMACE; Isele et al., 2015; Teicher & Parigger, 2015). The pediMACE consists of 45 dichotomous (yes/no) questions, measuring witnessed or self-experienced forms of childhood maltreatment throughout the lifetime. Only the seven items concerning abuse and the eight items concerning neglect by parental figures or caregiver were analyzed in this study (Supplementary Table 3). The pedMACE has been successfully validated with children in Tanzania (Isele et al., 2015). One sum score for lifetime experienced abuse types (range 0–7) and one for lifetime experienced neglect types (range 0–8) was computed. In the total sample, we interviewed 33 children with two independent raters to assess the interrater-reliability. Cohen's *k* interrater-reliability in the total sample was >0.99 (range=0.88–1).

#### Parental loss

Parental loss was assessed using one question: *Has your mother and/or your father passed away?* The age of the child at which the parental death occurred was assessed. In the event that both parents were deceased, the child's age at both times was identified.

#### Aggressive behavior

Aggressive behavior in the past 4 weeks was measured with the Reactive–Proactive Questionnaire (RPQ; Raine et al., 2006). Children are asked to rate the occurrence of different forms of aggression on a 3-point Likert scale with 0 (*never*), 1 (*sometimes*), and 2 (*often*). Following Hermenau et al. (2011) of the 23 items, one was not used and two were rephrased to be suitable for the living conditions of Tanzanian children. The RPQ previously achieved good psychometric properties in different cultural contexts (Fossati et al., 2009). The RPQ Score was computed by summing all item responses (range 0–44). In this sample, Cronbach's alpha coefficient was *α*=0.87 and Cohen's *k*-coefficient in the full sample was 0.99 (range=0.94–1).

#### Depression

Depressive symptoms in the past 4 weeks were assessed by means of the Children's Depression Inventory (CDI), which is a reliable clinical screening instrument assessing the severity of depressive symptoms in children and adolescents (Kovacs, 2001; Sitarenios & Kovacs, 1999). The CDI has 27 items with a 3-point Likert scale, ranging from 0 to 2 with higher values indicating more severe symptoms. Children are asked to pick one of three statements that best fits them. The CDI has been successfully used in Tanzania (Traube, Dukay, Kaaya, Reyes, & Mellins, 2010). The total severity score ranges from 0 to 54. In the current sample, Cronbach's alpha coefficient was *α*=0.77 and Cohen's *k*-coefficient in the full sample was 0.99 (range=0.93–1).

#### Internalizing and externalizing problems

The presence of internalizing and externalizing problems during the past 6 months was measured using the Strengths and Difficulties Questionnaire (SDQ) for children (Goodman, Meltzer, & Bailey, 1998). The instrument consists of 25 statements with answer categories ranging from 0 (*not true*), to 1 (*somewhat true*) or 2 (*certainly true*). The SDQ has good psychometric properties and has been widely used in African countries (Puffer et al., 2012; Wallis & Dukay, 2009). The sum of all items, excepting the ones for the prosocial behavior, represents the Total Difficulties Score (range 0–40). Cronbach's alpha coefficient in the present sample was *α*=0.63 and Cohen's *k*-coefficient in the full sample was 0.99 (range 0.94–1).

#### PTSD symptoms

PTSD symptoms in children were assessed by means of the UCLA PTSD Reaction Index for DSM-IV (Steinberg, Brymer, Decker, & Pynoos, 2004). The occurrence of each of the 17 DSM-IV symptoms within the last month is scored on a 5-point Likert scale ranging from 0 (*none of the time*) to 4 (*most of the time*). It has good psychometric properties and has been successfully used in African countries (Ellis, 2006). An overall PTSD severity score can be calculated by summing the scores for each symptom (of criteria B, C, D), which results in a maximum possible score of 68. Cronbach's alpha coefficient was *α*=0.90 and Cohen's *k-*coefficient in the full sample was 0.98 (range=0.82–1).

#### Perceived stigmatization

No standardized instruments currently exist to measure perceived stigmatization related to being an orphan. We measured perceived stigmatization using 10 purpose-built questions referring to orphans’ perception of being devalued by peers and teachers because of one's orphan status (Supplementary Table 4). Questions were phrased in the manner of, for example, *Is it true that other children think that orphans can be good friends?* or *Is it true that teachers think that orphans are more aggressive than other children?* The questionnaire aimed to systematically explore orphans’ awareness of being devalued by peers and teachers in situations similar to their daily experiences in a Tanzanian school. Item generation was inspired by orphans’ reports in qualitative research (e.g., Morantz et al., 2013). Orphans were asked to rate how much they agreed with the questions on a 5-point Likert scale ranging from 0 (*not at all*) to 4 (*very much*). Reversed statements were recoded. The sum of corresponding values of items was calculated (range: 0–40). The mean score in the group of orphans was 10.22 (SD=6.03, range: 1–23). Cronbach's alpha coefficient was *α*=0.62 and *k*-coefficient in the full sample was 0.98 (range=0.94–1). We tested the validity of our scale by correlating the sum score with stigma-related items of the SDQ and CDI. We found first hints for convergent and divergent validity (see Supplementary Table 5).

### Data analysis

To test hypotheses 1) and 2), we conducted two MANOVAs comparing orphans and non-orphans regarding their exposure to maltreatment and their psychological distress. Univariate analyses of variance (ANOVA) were then calculated. In order to avoid *α*-error inflation due to multiple testing, the Bonferroni-correction was applied. Following West, Finch and Curran (1995), all outcome variables were normally distributed except the UCLA score and neglect types for non-orphans. Violation of homogeneity was revealed by Levene's test between groups for CDI score, UCLA score and neglect types, and Box-M-Test for variance–covariance matrices in both MANOVAs. We used Pillai–Bartlett's trace for the MANOVAs since it is robust against violation of assumptions when sample sizes are equal (Bray & Maxwell, 1985). All analyses used a two-tailed *α*=0.05. For (M)ANOVAs, our metric for a small effect was *η* ^2^≥0.01, for a medium effect *η* ^2^≥0.06, and for a large effect *η* ^2^≥0.14.

To test hypotheses 3–5, we conducted multiple sequential regression analyses correlating neglect, abuse, perceived stigmatization, and the interaction of neglect and stigmatization both on the CDI score and the SDQ Total Difficulties Score. Correlates were z-standardized before analysis and significant interactions were analyzed via simple slopes according to Aiken and West (1991). Data sets were excluded listwise for data missing in the stigmatization score (*n*=10). All assumptions for linear regression models were tenable. Variance inflation factor (VIF) did not exceed 1.16, indicating no risk of multicollinearity. No outliers were detected. Residuals were normally distributed. Linearity, homoscedasticity, and independence were tenable. For multiple regression analyses, our metric for a small effect was *f* ^2^≥0.02, for a medium effect *f* ^2^≥0.15 and for a large effect *f* ^2^≥0.35. We used IBM SPSS Statistics Version 21 for data analyses. Using G*Power (Erdfelder, 1999), we calculated the power (1–β) of our analyses *post-hoc*.

## Results

Descriptive statistics are displayed in Table 1. Eighty-six orphans (97%) and 83 non-orphans (93%) reported having experienced at least one type of abuse by a caregiver throughout their lifetime. In total, 50 orphans (56%) and 28 (32%) non-orphans reported having experienced at least one type of neglect.

**Table 1:** Descriptive statistics and group comparisons between orphans and non-orphans

	Orphans^a^		Non-orphans^b^			
				
Outcome variables	*M*	SD	*M*	SD	*F* c	*η* ^2^
Neglect types	1.54	1.80	0.52	0.93	22.60**	0.11
Abuse types	3.46	1.50	3.39	1.66	0.08	<0.001
UCLA score	7.96	10.77	2.90	6.42	14.53**	0.08
CDI score	8.22	5.63	5.49	4.03	13.84**	0.07
RPQ score	9.89	6.39	7.49	5.31	7.39*	0.04
Total difficulties score	10.53	5.50	10.15	5.28	0.22	<0.001

UCLA, UCLA PTSD Reaction Index for DSM-IV; CDI, Children's Depression Inventory; RPQ, Reactive–Proactive Questionnaire; Total Difficulties Score, Strength and Difficulties Questionnaire. *M*, mean; SD, Standard deviation; *F*, test statistic of ANOVA; *η*^2^, effect size eta squared.

a *n*=89,

b *n*=89,

c *n*=178; *α*=0.0125 according to Bonferroni-correction.

* *p*≤0.01,

** *p*≤0.001.

### Maltreatment and psychological distress of orphans and non-orphans

There was a significant difference between orphans and non-orphans in the exposure to maltreatment types [*F*(2, 175)=11.78, *p<*0.001, *η* ^2^=0.12, power: 0.995]. Univariate ANOVAs revealed a significant difference with medium effect sizes for neglect types, whereas abuse types did not differ significantly (Table 1). There was also a significant group difference in the level of psychological distress [*F*(4, 173)=6.94, *p*<0.001, *η* ^2^=0.14, power: 0.998]. Univariate ANOVAs revealed significant differences with medium effect sizes in depression symptom severity, PTSD symptoms severity, and aggressive behavior (Table 1). No significant differences were found for internalizing and externalizing problems.

### Association of maltreatment and stigmatization to internalizing and externalizing problems

The first regression model including abuse and neglect accounted for 21% of the variability of the Total Difficulties Score (Table 2). Adding stigmatization improved the model significantly, *ΔR* ^2^=0.20, *F*(1, 75)=25.78, *p*<0.001, *f* ^2^=0.24. This second model explained 41% of the variance; the power of our analysis was >0.999. The main effect of neglect, abuse, and stigmatization correlated significantly positively with orphans’ internalizing and externalizing problems (Table 2). Adding the interaction term of neglect and stigmatization did not improve the model, *ΔR* ^2^=0.02, *F*(1, 74)=2.65, *p*=0.108, *f* ^2^=0.02.

**Table 2:** Sequential regression models on orphans’ psychological distress

	Outcome variables			
	
Predictor variables	*B*	*SE B*	β	*t*
**Internalizing and externalizing problems** a				
Step 1				
Abuse types	1.21	0.57	0.23	2.12*
Neglect types	1.59	0.49	0.35	3.26**
Step 2				
Abuse types	1.08	0.50	0.20	2.17*
Neglect types	1.47	0.42	0.33	3.48***
Stigmatization	2.46	0.48	0.45	5.08***
Step 3				
Abuse types	1.07	0.50	0.20	2.18*
Neglect types	1.40	0.42	0.31	3.33***
Stigmatization	2.20	0.51	0.40	4.36***
Neglect×stigmatization	0.66	0.41	0.15	1.63
**Depression symptom severity** b				
Step 1				
Abuse types	0.39	0.59	0.07	0.66
Neglect types	2.07	0.50	0.45	4.13***
Step 2				
Abuse types	0.30	0.56	0.06	0.54
Neglect types	1.99	0.48	0.43	4.18***
Stigmatization	1.70	0.54	0.30	3.12**
Step 3				
Abuse types	0.28	0.53	0.05	0.53
Neglect types	1.84	0.45	0.40	4.07***
Stigmatization	1.15	0.54	0.20	2.13*
Neglect×stigmatization	1.38	0.44	0.31	3.17**

*N*=79.

a SDQ Total Difficulties Score of orphans; final model (step 2): adj. *R*^2^=0.41, *F*(1, 75)=18.74, *p<*0.001, *f*^2^=0.68;

b CDI sum score of orphans; final model (step 3): adj. *R*^2^=0.36, *F*(4, 74)=12.16, *p*<0.001, *f*^2^=0.57. Abuse types, z-standardized sum score of experienced abuse types; Neglect types, z-standardized sum score of experienced abuse types; Stigmatization, z-standardized sum score of stigmatization items; *B*, unstandardized regression weight; *SE*, standard error; β, standardized regression weight; *t*, *t-*test statistics.

* *p*≤0.05.

** *p*≤0.01.

*** *p*≤0.001.

### Stigmatization as moderator of the relationship between neglect and depressive symptom severity

**Fig. 1:**
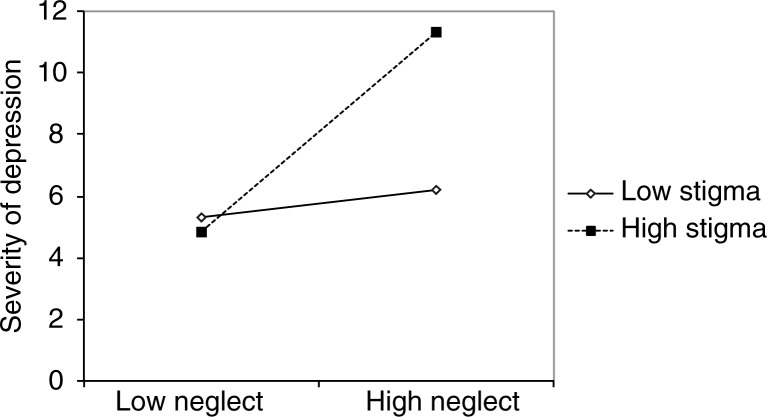
Moderating effect of perceived stigmatization (low: −1 SD; high: +1 SD) on the relationship between neglect types and depressive symptom severity.

The first regression model including abuse and neglect accounted for 21% of the variability of the CDI score (Table 2). Adding stigmatization improved the model significantly, *ΔR* ^2^=0.09, *F*(1, 75)=9.71, *p*=0.003, *f* ^2^=0.10. This second model explained 29% of the variance. Adding the interaction term of stigmatization and neglect improved the model again significantly, *ΔR* ^2^=0.08, *F*(1, 74)=10.05, *p*=0.002, *f* ^2^=0.09. The third model explained 36% of the variance in orphans’ severity of depression; the power of analysis was >0.999. The main effect of neglect and stigmatization as well as the interaction term of stigmatization and neglect correlated significantly positively with orphans’ depressive symptom severity (Table 2). Simple slope analysis indicated that the relationship between neglect and depression was significantly different at different values of perceived stigmatization (Fig. 1). For orphans reporting high levels of perceived stigmatization, (>1 SD above *M*) a significant, positive relationship between experienced types of neglect and severity of depression was found, *t*(4, 74)=5.43, *p* <0.001, whereas this relationship was not significant for orphans low in perceived stigmatization (>1 SD below *M*), *t*(4, 74)=0.69, *p*=0.492. Thus, perceived stigmatization moderated the neglect–depression relationship. In other words, the relationship is always positive but it is stronger if an orphan reports high levels of perceived stigmatization.

## Discussion

In the present study, we have systematically assessed exposure to neglect and abuse and compared orphans and matched non-orphans. We assessed different types of abuse and neglect; thus going beyond the common measurement of maltreatment via single items (Nyamukapa et al., 2010; Puffer et al., 2012). Most of the measurements used have been successfully implemented in African countries previously and achieved an acceptable to high reliability in the current study. Orphans reported experiencing more types of neglect throughout their lifetime than non-orphans. Yet, they did not differ regarding the number of reported abuse types. These findings were in concordance with our hypothesis and prior findings showed that neglect, but not necessarily abuse, is a factor specific to an orphan's exposure to maltreatment (Nichols et al., 2014). In Tanzania, corporal punishment and other abusive disciplinary practices are very common (Hecker et al., 2014). Concordantly, high rates of child abuse were found for both orphaned and non-orphaned children in the present sample.

Consistent with our hypothesis and previous results (Atwine et al., 2005; Puffer et al., 2012), orphans reported more depressive symptoms, PTSD symptoms, and aggressive behavior than non-orphans. The differences attained medium effect sizes. However, contradicting prior findings (Doku, 2009; Wild et al., 2011), no significant difference was found regarding internalizing and externalizing problems. However, previous findings have been inconsistent, with some studies in which both non-orphaned and orphaned children reported high rates of maltreatment also reporting no significant difference in behavior problems (Cluver & Gardner, 2006). Thus, the high rates of child abuse for both orphans and non-orphans in the current sample may partly explain these inconsistencies across studies.

In orphans, neglect, abuse, and perceived stigmatization correlated with internalizing and externalizing problems. This finding is consistent with prior evidence showing that child abuse is associated with children's externalizing and internalizing problems (Hecker et al., 2014) and that maltreatment and stigmatization were related to the psychological problems of orphans (Makame et al., 2002; Nyamukapa et al., 2010). Our results may be in support of the incremental effect of perceived stigmatization on an orphan's internalizing and externalizing problems (Cluver & Orkin, 2009), beyond the effect of maltreatment. Notably, our model explained more than 40% of the variance of the internalizing and externalizing problems, indicating large effect sizes.

Neglect and perceived stigmatization, but not abuse, correlated significantly with orphans’ depressive symptom severity. Similarly, Makame et al. (2002) documented that physical punishment did not relate to orphans’ internalizing problems, including depressive symptoms. The strong correlation between neglect and depressive symptoms indicates detrimental consequences of neglect on orphans’ mental health. In the current study, perceived stigmatization moderated the relationship between neglect and depressive symptoms. This relationship was particularly strong when an orphan reported high levels of perceived stigmatization. Our findings are consistent with the interactional model of risk factors for orphans’ depression (Cluver & Orkin, 2009): The more an orphan is aware of being stigmatized, the greater the relationship between adverse experiences and depression severity. Stigmatized orphans may have internalized others’ negative view of themselves and attribute negligent treatment to their (believed) inferiority as an orphan. Believing that they are a burden to society, they may in turn develop higher emotional problems. It will enhance our understanding of these mechanisms to incorporate various forms of stigmatization and other related constructs, such as self-esteem or quality of relationships, into future research models (Chi et al., 2014). Our model explained more than 35% of the variance of the depressive symptoms, indicating large effect sizes.

Thus far, only a few studies have systematically compared exposure rates of neglect and abuse between orphans and non-orphans, or identified factors relating to orphans’ psychological distress and assessed perceived stigmatization as an orphan. To better understand the impact of parental loss in sub-Saharan Africa, it is necessary to conduct longitudinal studies with greater sample size, testing comprehensive theoretical models of risk and protective factors on orphans’ mental health. More controlled studies on orphans’ exposure to neglect and abuse in low-income countries are worthwhile in order to create possibilities for meta-analysis on the issue.

The degree to which the results of the present study can be generalized is limited. First, the cross-sectional study design and the specific sample would not allow for establishing causality. However, evidence from longitudinal studies argue for the presence of long-term adverse effects of maltreatment on mental health (e.g., Widom et al., 2007). Our decision to include only non-orphaned children who had been living with their biological parents throughout their entire life may have influenced our findings as other non-orphaned children may suffer from the detrimental consequences of other adverse experiences (separation or divorce of parents). However, the average child in the full sample had been living with their parents throughout their entire life. The directional effect of perceived stigmatization on mental health, however, remains unclear (Mak et al., 2007). Depressed children may be more sensitive to negative attitudes about them and thus report higher levels of stigmatization (Cluver, Gardner, & Operario, 2008). Longitudinal and prospective study designs may help to invalidate this possible alternative explanation. The generalizability may be limited, but similar results have been obtained in different settings (Cluver et al., 2007). The orphan group was heterogeneous in regard to reasons for parental loss and care-arrangements. Furthermore, the presented data did not differentiate between the experiences of maltreatment before and after the death of one (or both) parent(s). It is possible that the experience of neglect was related to the parental illness or death. Nonetheless, sickness, dying, and death of a parent may affect the orphan's well-being and mental health. This being a realistic situation makes it difficult to control for all potential influences pre- and post- bereavement. Furthermore, our measure for perceived stigmatization has not yet been validated. Thus our findings have to be interpreted with caution. We measured children's general perception of being devalued or stigmatized because of their orphan status rather than experienced stigmatizing behavior by others. This may have underestimated the true effect. A potential bias, like social-desirability, can never be completely ruled out for self-report measures.

## Conclusions

Our findings indicate that parental loss is a stressor in children's development, putting orphans at higher risk of exposure to neglect and poor mental health. Particularly, perceived stigmatization as an orphan and neglect was negatively associated with orphans’ mental health. The relationship between neglect and depression was stronger if an orphan reported a high level of perceived stigmatization. Our results can be viewed in light of the growing number of orphans, poverty, overstretched care systems and lack of adequate child care policies in sub-Saharan Africa, resulting in poor caregiver ratios, untrained caregivers and lack of financial means to support the needs of vulnerable children (Hermenau et al., 2011; Hermenau, Kaltenbach, Mkinga, & Hecker, 2015). Yet the quality of care and support seems to be critical for children's development post-bereavement (Cerel, Fristad, Verducci, Weller, & Weller, 2006). Therefore, further research and culturally appropriate and evidence-based interventions are required for targeting the prevention of maltreatment and stigmatization of orphans.

## Supplementary Material

Neglect and perceived stigmatization impact psychological distress of orphans in Tanzania
