# Supplementary material for: Neglect and perceived stigmatization impact psychological distress of orphans in Tanzania
Source: Eur J Psychotraumatol. 2015 Nov 19;6:10.3402/ejpt.v6.28617. doi: 10.3402/ejpt.v6.28617 (PMC4654768; doi:10.3402/ejpt.v6.28617)
Supplement: Neglect and perceived stigmatization impact psychological distress of orphans in Tanzania [file EJPT-6-28617-s001.pdf]

Supplementary Information

Suppl. Table 1

Comparing half-orphans and full orphans regarding maltreatment and mental health

	Half-orphans (n = 73)		Full orphans (n = 16)		
Outcome Variables	*M*	*SD*	*M*	*SD*	F^a
Neglect types	1.45	1.68	1.94	2.32	0.95
Abuse types	3.63	1.29	2.69	1.99	5.44
UCLA score	8.55	11.36	5.31	7.21	1.19
CDI score	8.10	5.02	8.81	8.03	0.21
Total Difficulties Score	11.03	5.43	8.25	5.41	3.44
RPQ score	10.22	6.24	8.36	7.04	1.09

Note. $N = 89$; F (6, 82) = 3.03, $p = .01$; M = mean; SD = standard deviation; F= test statistic of ANOVA; UCLA = UCLA PTSD Reaction Index for DSM-IV; CDI = Children's Depression Inventory; RPQ = Reactive-Proactive Questionnaire; Total Difficulties Score = Strength and Difficulties Questionnaire.. $^aN = 89$; α = .0125 according to Bonferroni-correction.

Suppl. Table 2

Comparing orphans according to care-arrangement regarding maltreatment and mental health

	Orphans in foster care (n = 53)		Other orphans (n = 37)		
Outcome Variables	*M*	*SD*	*M*	*SD*	F^a
Neglect types	1.83	1.81	1.11	1.72	3.51
Abuse types	3.51	1.59	3.39	1.38	0.14
UCLA score	8.01	10.77	7.89	11.23	2.05
CDI score	8.92	6.20	7.19	4.56	0.003
Total Difficulties Score	11.11	5.67	9.67	5.19	1.50
RPQ score	10.40	6.18	9.14	6.70	0.83

Note. N = 89; F (6, 82) = 0.81, *p* = .565; M = mean; SD = standard deviation; F= test statistic of ANOVA; UCLA = UCLA PTSD Reaction Index for DSM-IV; CDI = Children's Depression Inventory; RPQ = Reactive-Proactive Questionnaire; Total Difficulties Score = Strength and Difficulties Questionnaire. [a]*N* = 89; α = .0125 according to Bonferroni-correction.

Suppl. Table 3

Occurrence of Abuse and Neglect Types for Orphans and Non-Orphans.

	Orphans *(n = 89)* % (*n*)	Non-orphans *(n = 89)* % (*n*)
Abuse Types		
1. Anybody called you names or said hurtful things such as calling you “fat”, “ugly” or “stupid”?	36 (32)	37 (33)
2. Anybody yelled/screamed at you?	75 (67)	80 (71)
3. Anybody locked you in a closet, attic, basement, garage, or another possibly narrow and dark place?	10 (9)	6 (5)
4. Anybody intentionally pushed, pinched, slapped, punched or kicked you?	62 (55)	60 (53)
5. Anybody spanked you with the palm of his/her hand on your buttocks, arms or legs?	54 (48)	54 (48)
6. Anybody spanked you with an object such as a strap, belt, brush, stick, tube, broom, wooden spoon, etc.?	82 (73)	83 (74)
7. Anybody hit you so hard that you were injured?	27 (24)	20 (18)
Neglect Types		
1. Was there a time in which both your mother and your father (or other main attachment figures/parental figures) did not try to understand your feelings, and were never there for you?	24 (21)	14 (12)
2. Was there a time in which neither your mother nor your father had time to talk to you, or were not interested in talking to you?	25 (22)	8 (7)

3. Did any family member (parent or parental figure) make you feel loved?	99 (88)	100 (89)
4. Did any family member take care of you?	98 (87)	100 (89)
5. Did any family member help you with your homework, or help you get ready for school?	94 (84)	99 (88)
6. Was there a time in which you did not have enough to eat?	17 (15)	3 (3)
7. Was there a time in which you had to wear dirty clothes?	8 (7)	5 (4)
8. Did a family member bring you to the doctor when necessary?	100 (89)	99 (88)

Suppl. Table 4

Perceived Stigmatization of Orphans (n = 79)

Is it true that…	M (SD)
… other children think that orphans get too much attention from teachers?	1.47 (1.59)
… other children think that orphans can be good friends? (r)	3.09 (1.28)
… other children think that orphans like fighting more than other children?	0.25 (0.69)
… other children think that orphans steal more than other children?	0.34 (0.80)
… other children think that orphans are like other children? (r)	2.08 (1.59)
… teachers think that orphans are more likely to lie than other children?	2.63 (1.63)
… teachers think that orphans are as intelligent as other children? (r)	0.33 (0.83)
… teachers think that orphans are more aggressive than other children?	0.48 (0.96)
… teachers think that orphans are just like other children? (r)	2.51 (1.67)

Note. Children were asked to rate how much they agreed with the questions on a 5-point Likert scale ranging from 0 (*not at all)* to 4 (*very much*). M = mean; SD = standard deviation.

Suppl. Table 5

Convergent and Divergent Validity of Perceived Stigmatization Score (n = 79)

	r
Convergent Validity	
I would rather be alone than with people of my age (SDQ 6).	.20†
I am often accused of lying or cheating (SDQ 18)	.25*
I get along better with adults than with people my own age (SDQ 23)	.27*
I feel alone all the time (CDI 20)	.26*
I never have fun at school (CDI 21)	.33**
Divergent Validity	
Other people of my age generally like me (SDQ 14).	-.24*
I like being with people (CDI 12; recoded)	-.27*

Note. Correlation of the Perceived Stigmatization Score with specific items of the SDQ and CDI. r = Pearson's correlation coefficient, CDI = Children's Depression Inventory; Total Difficulties Score = Strength and Difficulties Questionnaire. †$p \leq .10$. * $p \leq .05$. ** $p \leq .01$.
